# Shear wave elastography for extracranial carotid atherosclerotic plaques: technical principles and how to do it

**DOI:** 10.1590/1677-5449.202200822

**Published:** 2023-09-18

**Authors:** Pedro Luciano Mellucci, Matheus Bertanha, Rodrigo Gibin Jaldin, Winston Bonetti Yoshida, Marcone Lima Sobreira

**Affiliations:** 1 Universidade Estadual Paulista “Júlio de Mesquita Filho” - UNESP, Faculdade de Medicina de Botucatu - FMB, Botucatu, SP, Brasil.

**Keywords:** carotid artery diseases, carotid stenosis, Doppler ultrasonography, computer-assisted image interpretation, elasticity imaging techniques

## Abstract

In the wake of studies targeting atherosclerotic plaques and searching for quantifiable variables that contribute additional information to therapeutic decision-making, plaque assessment using Shear Wave Elastography (SWE) is emerging as a reproducible and promising alternative. We used a single Logiq S8 device (General Electric, Boston, Massachusetts, United States) with an 8.5-11MHz multifrequency linear transducer at 10MHz in longitudinal section. We considered relevant criteria for image acquisition: adequate longitudinal insonation, differentiation of the intima-media complex, delineation of proximal and distal tunica adventitia and the vascular lumen, good visualization of the atherosclerotic plaque, cardiac cycle in ventricular diastole, and absence of incongruous changes. SWE is an emerging and extremely promising method for assessment of carotid plaques that may contribute to therapeutic decision-making based on characteristics related to the atherosclerotic plaque, with inter-device and inter-examiner reproducibility.

## INTRODUCTION

Cerebral vascular accident (stroke) caused by carotid stenosis of extracranial origin is a subject that has been extensively studied over recent decades, with well-defined causal correlations,^1^ and randomized studies with large populations of symptomatic^2-4^ and asymptomatic patients.^5,6^ Conclusions with regard to the best treatment for these patients have been proposed with satisfactory evidence levels and well-defined therapeutic flow charts have been published by the European Society for Vascular Surgery (ESVS)^7^ and the Society for Vascular Surgery (SVS) consensus groups.^8^ However, objective analysis of atherosclerotic plaques for decision making is still to a great extent restricted to assessing the degree of stenosis present.

It is postulated that the risk of stroke is also intimately related to the morphological characteristics of the atherosclerotic plaque. The Oxford Plaque Study^9^ observed ulcerations in up to 58.1% of symptomatic carotid plaques and hemorrhages and inflammation were present in up to 64.6% and 66.8% of cases, respectively.

Using data from the North American Symptomatic Carotid Endarterectomy Trial (NASCET),^3^ it was observed that objectives parameters correlated increased risk of development of stroke in relation to the area of the atherosclerotic plaque, presence of “discrete white areas” (DWAs), and grayscale median (GSM) values less than 30, with up to 70% stroke risk at 5 years when all three factors were present.^10^

**Figure 1:**
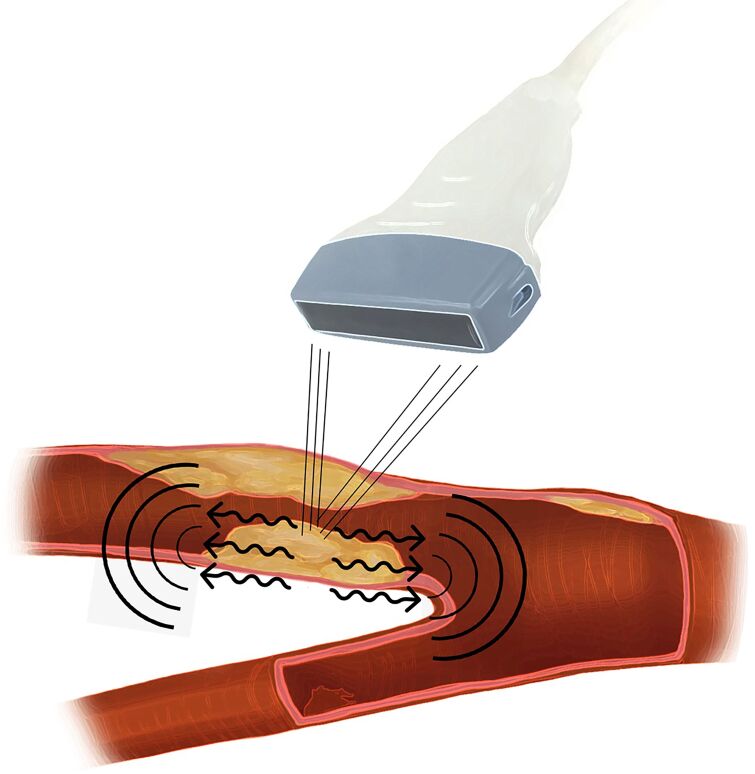
Schematic image illustrating the shear wave and its propagation perpendicular to the insonated tissue.

In the wake of studies targeting atherosclerotic plaques and seeking quantifiable variables that contribute additional information to therapeutic decision-making, assessment using elastography is emerging as a reproducible and promising alternative.^11^ Ultrasonography incorporating shear wave elastography (SWE) is a recent technique in which a powerful acoustic wave is emitted by an ultrasound probe, inducing a perpendicular wave after hitting the target tissue (Figure 1). The shear wave that propagates perpendicular to the original wave is measured and is proportional to the hardness of the tissue; a correlation that can be expressed as Young’s modulus.^12^

The objective of this article is to describe the method used to acquire values for carotid atherosclerotic plaque rigidity using vascular echography in conjunction with the SWE method. While initial studies with small samples^13-22^ have proposed using elastography in the carotid region, the lack of a detailed methodological description merits publication of a technical paper to provide a guide for future studies of the subject.

## METHODS

### Image acquisition

Ultrasonographic images were acquired in B-mode by two ultrasonographers, both board certified by the Brazilian College of Radiologists (Colégio Brasileiro de Radiologistas – CBR) and the Brazilian Society of Angiology and Vascular Surgery (Sociedade Brasileira de Angiologia e de Cirurgia Vascular – SBACV), using a single Logiq S8 (General Electric, Boston, Massachusetts, United States) ultrasound machine with an 8.5-11 MHz multifrequency linear transducer at 10 MHz in longitudinal section.

**Figure 2:**
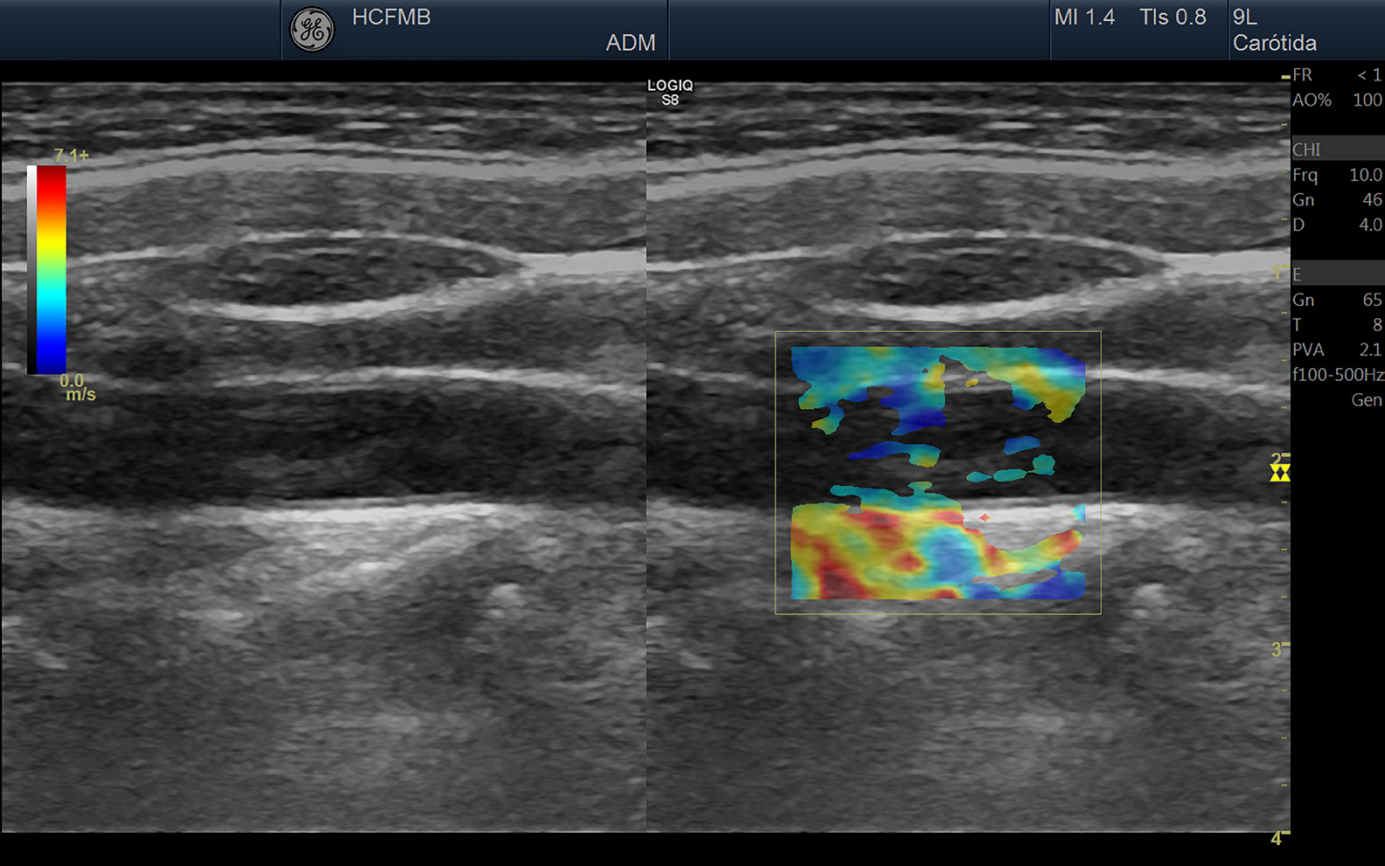
Image acquired with elastography (on the right), where the vascular lumen is either outside of the color map or tending to dark blue colors (lower stiffness), while the tunica adventitia tends to red colors (higher stiffness).

Dual mode (displaying two images) was used for all assessments so that the examiner had the B-mode image available on the left, to maintain insonation steady over the area of interest, with the elastography processing image on the right. The area selected for elastography processing had to include the anterior and posterior tunica adventitia and the vascular lumen, with the objective of specifying adequate frames that show hardness tending to zero in the arterial lumen (blood) and satisfactorily delimiting the vascular wall (Figure 2).

The number of foci, focal distance, time gain compensation (TGC), and gain were not standardized for B mode, color mode, or Doppler mode when performing elastography. It should be considered that the method is independent of B-mode gain, unlike GSM assessment, which requires standardized gain to analyze pixels. For elastography, the priority is to obtain images that maximize the morphological aspects of the atherosclerotic plaque.

It is also important to point out that use of elastography is still limited to equipment with high processing power that has the dedicated software onboard. This differentiates it from GSM, which can be effectively performed using any ultrasound machine, but must be analyzed after post-processing.

### Processing power and frames-per-second (FPS)

We chose to acquire images for a minimum of 10 seconds, because of the considerable drop in frame rate (expressed in FPS) when elastography is performed, since it puts a high demand on the machine’s processing power, and also because of vascular mobility, which was a determinant factor in the technical difficulty of performing the examination in the carotid region. The number of frames acquired before a satisfactory image is achieved is still subjective and may be broadly determined by the processing power of the equipment being used. It is expected that the number of frames in a given interval of time will be proportional to the machine’s processing power and there will possibly be minor implications secondary to the cardiac cycle.

### Cardiac cycle and Young’s modulus

**Figure 3:**
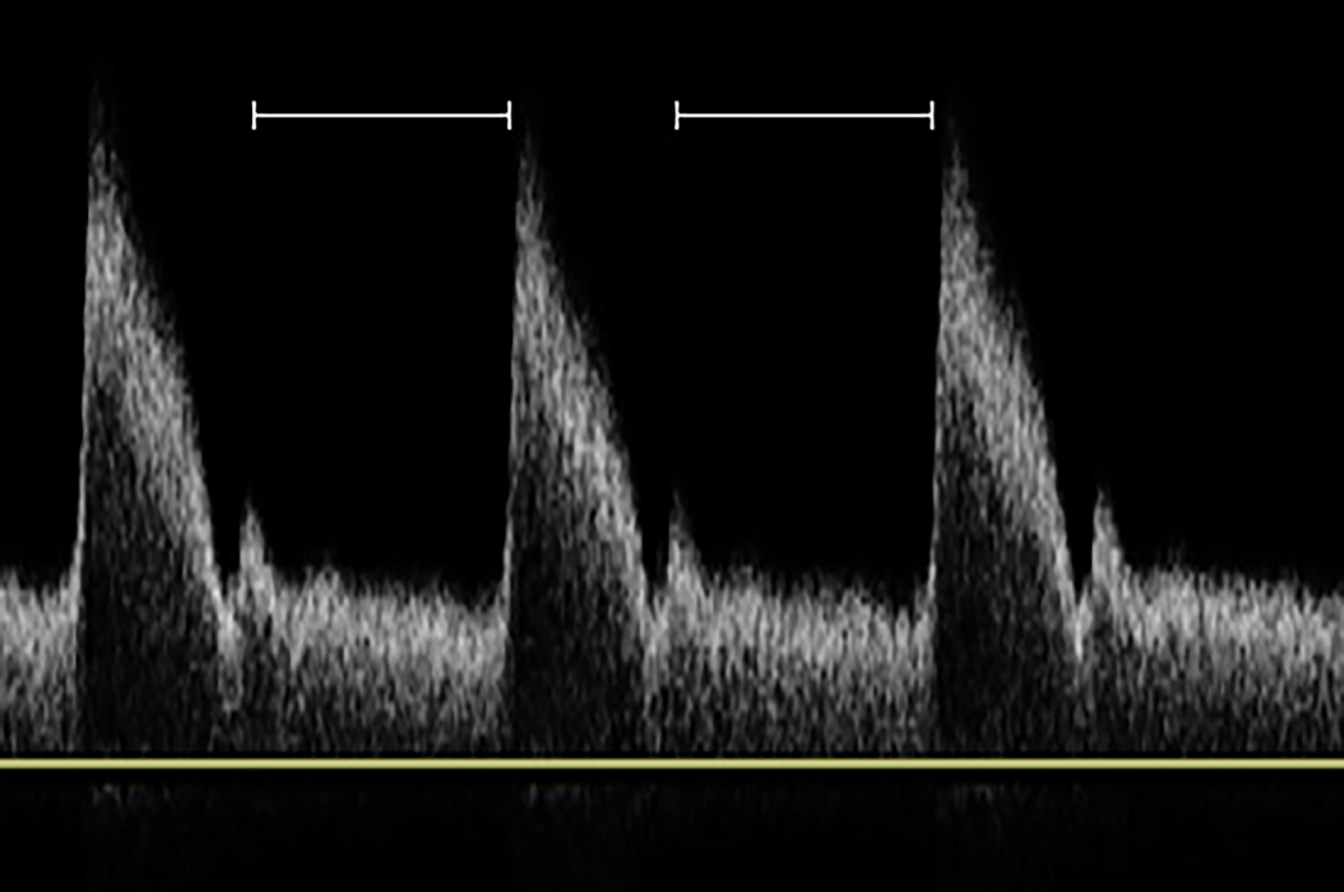
Ventricular diastole in the arterial pulse wave.

**Figure 4:**
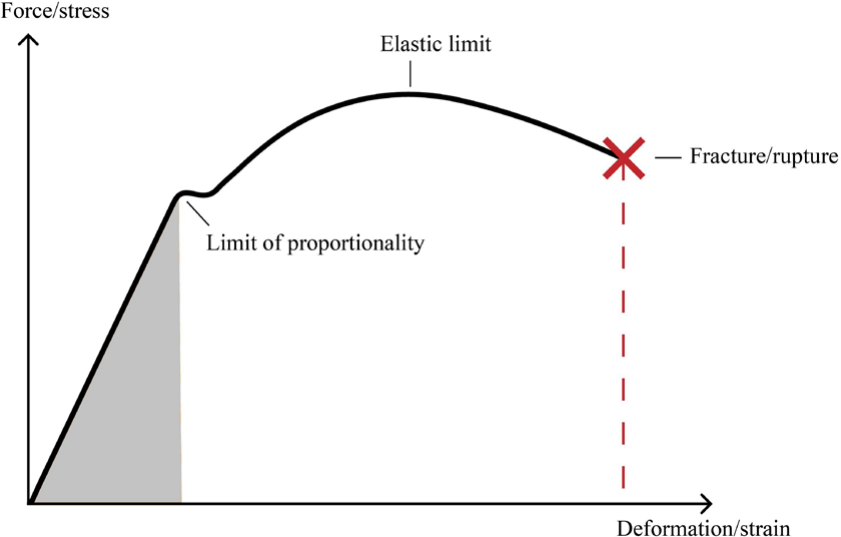
Graph illustrating the relationship between force exerted on a tissue (stress), represented by σ, and its deformation (strain), represented by ε, according to Young’s modulus.

With relation to the cardiac and vascular cycles, we acquired images in consonance with ventricular diastole (Figure 3), which accounts for the greater part of the cardiac cycle and regression of the vascular wall to its original morphology after the pulse wave has passed. We chose to take measurements during this phase of the cardiac cycle because of the greater ΔT (time) for processing the shear waves (invariably dependent on the machine used) and considering Young’s modulus (E), which measures the tension of a solid when a force is applied and is given by the formula E = σ/ε, where σ is the force applied to a given area (stress) and ε is the axial deformation (strain) of the solid, which, simplistically, indicates the increase in artery wall hardness as the pulse wave passes (Figure 4).

Arterial systole (or ventricular diastole) can be observed indirectly by the reduction of arterial diameter on B-mode (demonstrating the importance of conducting the examination with dual mode if possible), with no need for an electrocardiogram to be fitted during the examination. According to Young’s modulus, during the passage of the pulse wave, deformation of the vascular wall is maximal and directly proportional to the force exerted on it by the blood column.

### Measurement of the region of interest

**Figure 5:**
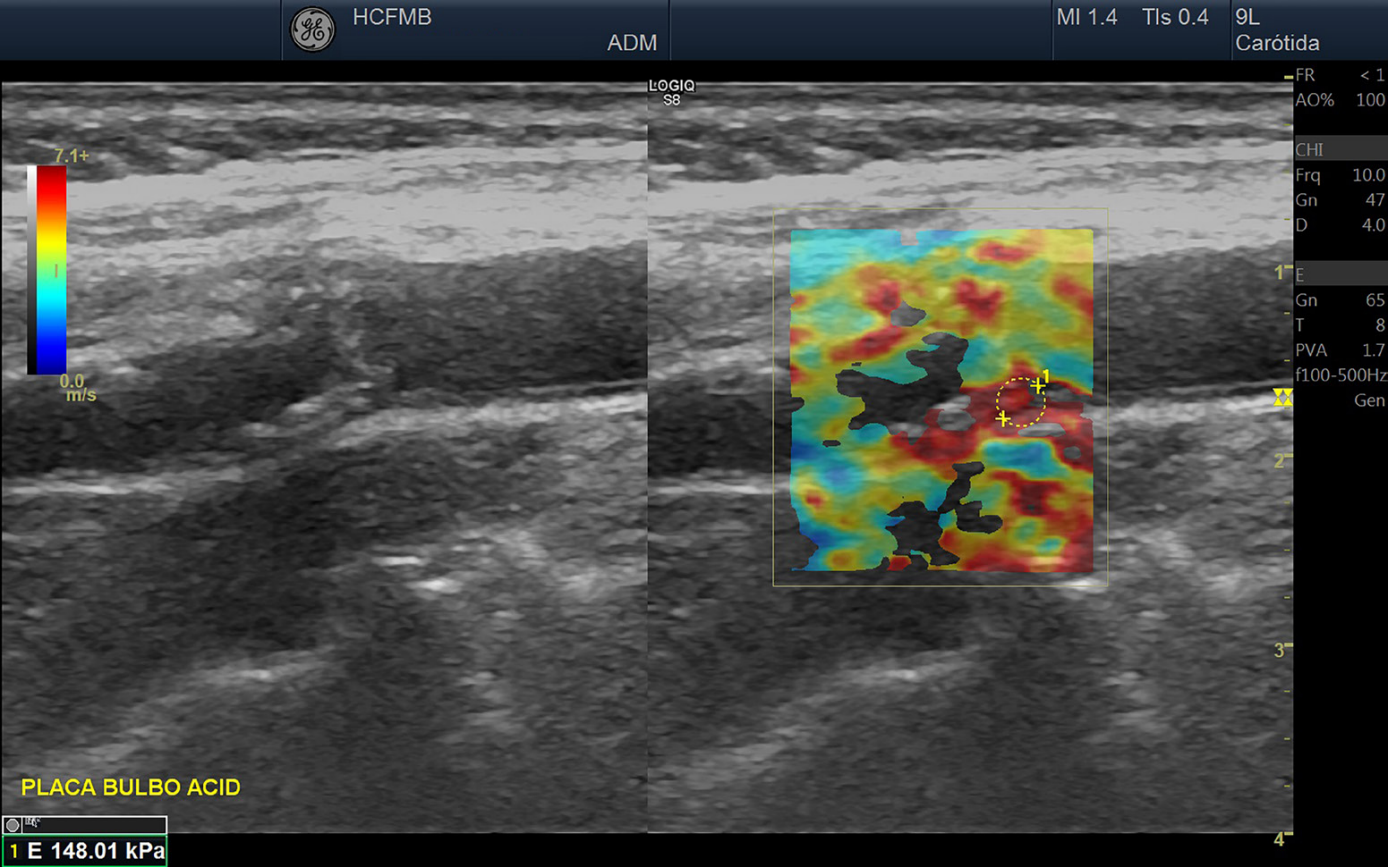
Image acquisition in dual mode (double window), with elastography on the right, showing selection of the region analyzed and the criteria for good image adequacy.

We assess the region of interest within the atherosclerotic plaque, excluding the posterior tunica adventitia by measuring within a circular area, obtaining a result in kilopascals (kPa), the international unit of pressure, from a single frame, as long as it meets a series of adequacy criteria (Table 1 and Figure 5). The image is saved in dual mode and the tension at the region of interest is noted in a report, together with the morphological characteristics of the atherosclerotic plaque studied.

**Table 1:** Criteria adopted by the Vascular and Endovascular Surgery Service at the Hospital das Clínicas of the Faculdade de Medicina de Botucatu (FMB-UNESP) for acquisition of good images for elastography of atherosclerotic carotid plaques.

Adequate longitudinal insonation
Adequate differentiation of the intima-media complex
Delineation of the proximal and distal tunica adventitia (kPa > 7.1)
Delineation of the vascular lumen (kPa < 7.1)
Delineation of the atherosclerotic plaque
Ventricular diastole
Absence of incongruent changes

### Ethics

This study does not present any data that could identify patients, comprising a technical description and a review of the literature. However, the methodology described was part of a pilot study, the protocol for which was approved by the Ethics Committee at our institution, with Ethics Appraisal Submission Certificate number 36750720.4.0000.5411, emitted by the Research Ethics Committee at the Universidade Estadual Paulista, Faculdade de Medicina de Botucatu (UNESP-FMB), consolidated opinion number 4.296.479.

## DISCUSSION

Elastography is not yet an accessible method, especially not in developing countries, to a great extent because of the costs involved in performing the technique, usually employing high end equipment that is very often limited to research centers. The models employed most recently include the Supersonic Aiexplorer (Hologic, Marlborough, Massachusetts, United States),^13-19^ Logiq E9 (General Electric, Boston, Massachusetts, United States)^20^ e Aplio 500 (Toshiba, Tokyo, Japan).^21,22^ These machines’ high processing power is needed to obtain a high FPS while performing the technique and to obtain images quickly, between two pulse waves, avoiding artifacts caused by movement of the vascular tissue. In addition to the technical difficulty of obtaining images free from artifacts caused by movement of tissues, use of suboptimal equipment for the examination can result in elevated subjectivity of image acquisition.

To date, the majority of in vivo studies have performed measurements related to the characteristics of atherosclerotic plaques in longitudinal section.^13-22^ In 2020, Marlevi et al.^13^ performed a study comparing SWE and magnetic resonance imaging (MRI), findings that results differed between cross-sectional and longitudinal images. Considering the circular region of interest and the greater exposure of the atherosclerotic plaque, the longitudinal plane appears preferable for these measurements.

When compared with other imaging methods such as MRI and computed tomography (CT), SWE was able to identify vulnerable plaques, validated by MRI,^13^ and was more sensitive but less specific than CT.^15^ With regard to the comparison between SWE and GSM, the former offers the possible advantage of being less dependent on B-mode standardization to obtain the value measured, improving inter-examiner and inter-apparatus reproducibility,^18-20,23^ although the GSM measure does allow for grayscale standardization with image post-processing. Ramnarine et al.^22^ conducted a study with 54 atherosclerotic plaques, observing that SWE achieved a better predictive value than GSM for identification of symptomatic plaques.^22^

To date, SWE has demonstrated good reproducibility for the carotid region.^18-20^ It also offers a statistically significant correlation for identification of symptomatic and vulnerable plaques.^18-22^ Since this is a novel and emerging method, its adoption is still restricted to research centers and the methodology still needs to be standardized.

## CONCLUSIONS

Shear wave elastography is an emerging method with promise in the context of assessment of carotid plaques and in the future, it may contribute to therapeutic decision making based on characteristics related to the atherosclerotic plaque, with inter-device and inter-examiner reproducibility. As more studies are conducted, it is possible that relevant prognostic factors can be extracted from plaque stiffness assessments, particularly considering that plaques with a lipid core and intra-plaque hemorrhages increase the likelihood of thrombotic events and these have much lower stiffness than calcium or fibrosis. However, standardized methodology for performing elastography of carotid plaques has not yet been defined, which has contributed to difficulties with adoption of the method.

Cutting-edge vascular ultrasonography equipment is needed to conduct examinations free from major examiner subjectivity, because of the considerable reduction in FPS related to the examination’s high processing demands and the artifacts related to the pulse wave.

With constant technological and scientific development, it is possible that elastography during carotid plaque echography will become common, considering that it only takes a few seconds and that the machine itself can calculate the stiffness of the atherosclerotic plaque in a quantitative manner without the need for analysis after post-processing, which is an important limitation of other methods of plaque assessment, such as GSM.
