# Supplementary material for: Shear wave elastography for extracranial carotid atherosclerotic plaques: technical principles and how to do it
Source: J Vasc Bras. 2023 Sep 18;22:e20220082. doi: 10.1590/1677-5449.202200822 (PMC10545232; doi:10.1590/1677-5449.202200822)
Supplement: Translation

# Translation: Elastografia por cisalhamento (*shear wave*) para placas ateroscleróticas carotídeas extracranianas: princípios técnicos e como realizar

**DOI:** 10.1590/1677-5449.202200821

**Authors:** Pedro Luciano Mellucci, Matheus Bertanha, Rodrigo Gibin Jaldin, Winston Bonetti Yoshida, Marcone Lima Sobreira

**Affiliations:** 1 Universidade Estadual Paulista “Júlio de Mesquita Filho” - UNESP, Faculdade de Medicina de Botucatu - FMB, Botucatu, SP, Brasil.

**Keywords:** doenças das artérias carótidas, estenose das carótidas, ultrassonografia Doppler, interpretação de imagem assistida por computador, técnicas de imagem por elasticidade

## Abstract

Na esteira de estudos direcionados à placa aterosclerótica e em busca de variáveis quantificáveis que adicionem informações à tomada de decisão terapêutica, a avaliação a partir de elastografia *shear wave* (SWE) se apresenta como alternativa reprodutível e promissora. Utilizamos um único aparelho Logiq S8 (General Electric, Boston, Massachusetts, Estados Unidos) com um transdutor linear multifrequencial 8,5-11 MHz em 10 MHz em corte longitudinal. Consideramos critérios relevantes para a aquisição de imagem: adequada insonação longitudinal, diferenciação do complexo médio-intimal, delineamento de túnicas adventícias proximal e distal, lúmen vascular, boa visualização da placa aterosclerótica, ciclo em diástole ventricular e ausência de alterações incongruentes. A SWE é um método emergente e extremamente promissor no contexto da avaliação de placas carotídeas, podendo contribuir no futuro para a tomada de decisão terapêutica baseada em características relativas à placa aterosclerótica de forma reprodutível entre aparelhos e examinadores.

## INTRODUÇÃO

O acidente vascular cerebral (AVC) oriundo da estenose carotídea de origem extracraniana foi um assunto extensamente estudado durante as últimas décadas, com correlações causais bem definidas^1^ e estudos randomizados em grandes populações de pacientes sintomáticos^2-4^ e assintomáticos^5,6^ . Conclusões acerca da melhor terapêutica para esses pacientes foram propostas com nível de evidência satisfatório, com fluxogramas terapêuticos bem definidos pelos consensos da European Society for Vascular Surgery (ESVS)^7^ e da Society for Vascular Surgery (SVS)^8^ ; entretanto, a análise objetiva da placa aterosclerótica para tomada de decisão ainda é, em grande parte, restrita ao grau de estenose apresentado.

Postula-se que o risco do AVC também esteja intimamente relacionado a características morfológicas da placa aterosclerótica. No trabalho desenvolvido pelo Oxford Plaque Study^9^ , ulcerações foram observadas em até 58,1% das placas carotídeas sintomáticas, e hemorragias e inflamações estiveram presentes em até 64,6% e 66,8% dos casos, respectivamente.

Utilizando dados oriundos do grupo North American Symptomatic Carotid Endarterectomy Trial (NASCET)^3^ , observaram-se parâmetros objetivos correlacionados a risco aumentado de desenvolvimento do AVC em relação à área da placa aterosclerótica, presença de “áreas brancas discretas” (DWAs, do inglês *discrete white areas*) e valores de mediana de escala de cinza (GSM, do inglês *greyscale median*) inferiores a 30, com riscos de AVC em 5 anos chegando a até 70% na presença dos três fatores^10^ .

Na esteira de estudos direcionados à placa aterosclerótica e em busca de variáveis quantificáveis que adicionem informações para a tomada de decisão terapêutica, a avaliação a partir de elastografia se apresenta como alternativa reprodutível e promissora^11^ . A ultrassonografia associada a elastografia por cisalhamento (SWE, do inglês *shear wave elastography*) é uma técnica recente, em que uma onda acústica potente é emitida pela sonda ecográfica, induzindo uma onda perpendicular após chocar-se com o tecido objetivado (Figura 1). A onda de cisalhamento que se propaga perpendicularmente à onda original é aferida, sendo proporcional à dureza do tecido, correlação representada pelo módulo de Young^12^ .

**Figura 1:** Imagem esquemática demonstrando a onda de cisalhamento e sua propagação perpendicular ao tecido insonado.

Este artigo tem como objetivo descrever o método utilizado para aquisição de valores de rigidez de placas ateroscleróticas carotídeas a partir da ecografia vascular associada ao método de SWE. Embora estudos iniciais com pequena amostragem^13-22^ tenham se proposto à realização da elastografia no território carotídeo, a ausência de descrição metodológica detalhada justifica uma nota técnica para servir como guia para os futuros estudos acerca do tema.

## MÉTODOS

### Aquisição da imagem

Imagens ultrassonográficas em modo B foram obtidas por dois ultrassonografistas titulados pelo Colégio Brasileiro de Radiologistas (CBR) e pela Sociedade Brasileira de Angiologia e de Cirurgia Vascular (SBACV), utilizando um único aparelho Logiq S8 com um transdutor linear multifrequencial 8,5-11 MHz em 10 MHz em corte longitudinal.

O modo dual (apresentação da imagem em duas janelas) foi utilizado para todas as avaliações, de modo que o examinador obtivesse acesso à imagem em modo B para manutenção de insonação regular sobre a área de interesse à esquerda e o processamento da elastografia à direita. A área de processamento da elastografia deve englobar a túnica adventícia anteriormente e posteriormente, além do lúmen vascular, com o objetivo de caracterizar quadros adequados que demonstrem uma dureza que tende a zero no lúmen arterial (sangue) e delimite a parede vascular satisfatoriamente (Figura 2).

**Figura 2:** Imagem adquirida com elastografia (à direita), onde o lúmen vascular apresenta-se fora do mapa de cores ou tendendo a cores azuis escuras (rigidez menor), enquanto a túnica adventícia apresenta cores tendendo ao vermelho (rigidez maior).

O número de focos, a distância focal, a *time gain compensation* (TGC) e o ganho dos modos B, colorido ou Doppler não foram padronizados para a realização da elastografia. Deve-se considerar a independência do método em relação ao ganho do modo B, diferentemente da avaliação de GSM, que necessita de uma padronização de ganho para a avaliação de *pixels*. Dessa forma, deve-se priorizar a obtenção de imagens que maximizem os aspectos morfológicos da placa aterosclerótica.

É importante salientar que a elastografia ainda é um método restrito para uso em aparelhos com alto poder de processamento e que contem com *software* próprio, presente na máquina. Essa é uma especificação que difere da avaliação da GSM, que pode ser realizada de forma eficaz em qualquer aparelho, mas que depende de uma avaliação a partir de pós-processamento.

### Poder de processamento e quadros por segundo

Optamos pela aquisição de imagens por um período mínimo de 10 segundos, considerando a queda expressiva do *frame rate* (quadros por segundo [FPS]) durante a realização da elastografia, que recruta um expressivo poder de processamento do aparelho, além da mobilidade vascular, que foi fator determinante para a dificuldade técnica do exame no território. A quantidade de quadros obtidos até aquisição de uma imagem satisfatória ainda é subjetiva e pode ser largamente determinada pelo poder de processamento do aparelho em questão. Espera-se uma quantidade de quadros em um intervalo de tempo proporcional ao processamento do aparelho, possivelmente com menores implicações secundárias ao ciclo cardíaco.

### Ciclo cardíaco e módulo de Young

Em relação ao ciclo cardíaco e vascular, realizamos a aquisição de imagens em consonância com a diástole ventricular (Figura 3), que representa a maior parte do ciclo cardíaco e regresso da parede vascular à sua morfologia original após a passagem da onda de pulso. Optamos pela realização da medida nesta fase do ciclo cardíaco pelo maior ΔT (tempo) para processamento das ondas de cisalhamento (invariavelmente dependente do aparelho utilizado) e considerando o módulo E de Young, que mede a tensão de um sólido quando uma força é aplicada, determinado pela formula E = σ/ε, em que σ representa a força imprimida sobre uma determinada área (*stress*) e ε representa a deformação axial do sólido (*strain*), que, simplificadamente, aponta para um aumento da dureza da parede arterial durante a passagem da onda de pulso (Figura 4).

**Figura 3:** Demonstração da diástole ventricular na onda de pulso arterial.

**Figura 4:** Gráfico demonstrando a relação entre força imprimida sobre um tecido (*stress*), representada por σ, e a sua deformação (*strain*), representada por ε, de acordo com o módulo de Young.

A observação da sístole arterial (ou diástole ventricular) pode ser feita de forma indireta pela redução do diâmetro arterial ao modo B (observa-se a importância do exame em modo *dual* se possível), sem a necessidade de eletrocardiograma acoplado ao exame. De acordo com o módulo de Young, durante a passagem da onda de pulso, a deformação da parede vascular é máxima e diretamente proporcional à força imprimida sobre ela pela coluna de sangue.

### Aferição da área de interesse

Avaliamos a área de interesse dentro da placa aterosclerótica, excluindo a túnica adventícia posterior a partir de medida em uma área circular, com resultado obtido em kilopascais (kPa), unidade internacional de tensão, a partir de quadro único, desde que preenchida uma série de critérios para adequação (Tabela 1 e Figura 5). A imagem em modo dual é salva, e a tensão da área de interesse é descrita em laudo juntamente às características morfológicas da placa aterosclerótica estudada.

**Tabela 1:** Critérios adotados no Serviço de Cirurgia Vascular e Endovascular do Hospital das Clínicas da Faculdade de Medicina de Botucatu (FMB-UNESP) para boa aquisição de imagem para elastografia de placa aterosclerótica carotídea.

Adequada insonação longitudinal
Adequada diferenciação do complexo médio-intimal
Delineamento das túnicas adventícias proximal e distal (kPa > 7,1)
Delineamento do lúmen vascular (kPa < 7,1)
Delineamento da placa aterosclerótica
Diástole ventricular
Ausência de alterações incongruentes

**Figura 5:** Aquisição de imagem em modo dual (tela dupla), com elastografia à direita, demonstrando delimitação de área analisada e critérios para boa adequação da imagem.

### Ética

O presente estudo não apresenta dados que identifiquem pacientes, com representação técnica e revisão de literatura. Entretanto, a metodologia exposta faz parte de estudo-piloto, cujo protocolo foi aprovado pelo Comitê de Ética de nossa instituição, com Certificado de Apresentação de Apreciação Ética número 36750720.4.0000.5411, Comitê de Ética em Pesquisa (CEP) da Universidade Estadual Paulista, Faculdade de Medicina de Botucatu (UNESP-FMB), sob o registro de parecer consubstanciado 4.296.479.

## DISCUSSÃO

A elastografia ainda é um método pouco acessível, especialmente em países em desenvolvimento, em grande parte devido ao custo empregado na realização da técnica, usualmente em aparelhos top de linha muitas vezes restritos a centros de pesquisa. Entre os aparelhos mais recorrentemente utilizados, encontramos Supersonic Aiexplorer (Hologic, Marlborough, Massachusetts, Estados Unidos)^13-19^ , Logiq E9 (General Electric, Boston, Massachusetts, Estados Unidos)^20^ e Aplio 500 (Toshiba, Tokio, Japão)^21,22^ . O elevado poder de processamento desses aparelhos é relevante para a obtenção de FPS elevados durante a realização da técnica e para a obtenção de imagens de forma ágil entre duas ondas de pulso, evitando artefatos causados pela mobilização do tecido vascular. Além da dificuldade técnica para obter imagens livres de artefato causado por mobilização tecidual, o uso de aparelhos não otimizados para o exame pode levar a uma elevada subjetividade na obtenção das imagens.

Até o momento, a maior parte estudos *in vivo* realizou a medida das características relativas à placa aterosclerótica em cortes longitudinais^13-22^ . Em 2020, Marlevi et al.^13^ , em estudo comparativo entre SWE e ressonância magnética (RNM), identificaram achados que diferiram entre imagens em plano transverso e longitudinal. Considerando o delineamento circular da área de aferição e a maior exposição da placa aterosclerótica, o plano longitudinal parece ser preferível para a medida.

Quando comparada com outros métodos de imagem como RNM e a tomografia computadorizada (TC), a SWE foi capaz de identificar placas vulneráveis, validadas por RNM^13^ , sendo mais sensível e menos específica do que a TC^15^ . Quanto à comparação entre SWE e GSM, a primeira apresenta a possível vantagem de depender menos de padronização do modo B para se obter o valor medido, o que torna a avaliação entre examinadores e aparelhos mais reprodutível^18-20,23^ , embora a medida de GSM permita a padronização da escala de cinza no pós-processamento de imagem. Em estudo com 54 placas ateroscleróticas, Ramnarine et al.^22^ observaram que a SWE apresentou um valor preditivo superior à GSM na identificação de placas sintomáticas.

Até o momento, a SWE tem apresentado boa reprodutibilidade no território carotídeo^18-20^ . Apresenta, ainda, correlação estatisticamente significativa na identificação de placas sintomáticas e vulneráveis^18-22^ . Como um método novo e emergente, a sua difusão ainda é restrita a centros de pesquisa, e sua metodologia ainda precisa ser padronizada.

## CONCLUSÃO

A SWE é um método emergente e promissor no contexto da avaliação de placas carotídeas, podendo contribuir no futuro para a tomada de decisão terapêutica baseada em características relativas à placa aterosclerótica de forma reprodutível entre aparelhos e examinadores. Com o desenvolvimento de mais estudos, é possível que fatores prognósticos relevantes possam ser extraídos a partir da avaliação da rigidez da placa, em especial se considerando que placas de núcleo lipídico e hemorragias intraplacas aumentam a chance de eventos trombóticos e que estes apresentam uma rigidez muito inferior ao cálcio ou fibrose. Uma metodologia padronizada para a realização da elastografia em placas carotídeas, entretanto, ainda não foi definida, o que contribui para a dificuldade na disseminação do método.

Aparelhos de ultrassonografia vascular de ponta ainda são necessários para a realização de um exame livre de maior subjetividade do examinador devido à expressiva queda dos FPS relativa à alta demanda de processamento do exame e artefatos relativos à onda de pulso.

Com a constante evolução tecnológica e científica, é possível que a elastografia se torne uma realidade na avaliação da ecografia da placa carotídea, considerando que sua realização leva apenas alguns segundos e que o próprio aparelho utilizado pode calcular a rigidez da placa aterosclerótica de forma quantitativa sem a necessidade de avaliação a partir de pós-processamento, o que é um limitante importante de outros métodos de avaliação da placa tal qual a GSM.
